# Virtual reality for patient informed consent in skull base tumors and intracranial vascular pathologies: A pilot study

**DOI:** 10.1007/s00701-024-06355-w

**Published:** 2024-11-15

**Authors:** Emilia Westarp, Attill Saemann, Marek Zelechovski, Balazs Faludi, Philippe Cattin, Jehuda Soleman, Raphael Guzman

**Affiliations:** 1 Department of Neurosurgery, University Hospital Basel, University of Basel, Spitalstrasse 21, 4031 Basel, Switzerland; 2 Department of Biomedical Engineering, University of Basel, Allschwil, Switzerland; 3 Faculty of Medicine, University of Basel, Basel, Switzerland

**Keywords:** Virtual reality, Patient informed consent, Neurosurgery, Subjective patient comprehension

## Abstract

**Purpose:**

With the growing demand for shared decision-making and patient-centered care, optimal informed consent (IC) has gained relevance. Virtual reality (VR) has seen significant technological advancements, and its medical applications currently include surgical planning and medical education. This pilot study investigates the feasibility of VR-enhanced informed consent (VR-IC) in neurosurgery to improve preoperative IC and patient satisfaction.

**Methods:**

We included patients aged 18 to 75 years who were scheduled for skull base meningioma or brain aneurysm surgery between May and December 2023. Exclusion criteria were visual/auditory impairments and severe cognitive/psychiatric disorders. Patients received standard IC followed by VR-IC using patient-specific VR models of their pathology. After an initial demonstration by the surgeon, the patients used the VR station independently. A questionnaire with 18 questions on a 5-point Likert scale assessed the subjective impression of VR-IC.

**Results:**

Ten patients participated in the study, with six (60%) undergoing aneurysm clipping and four (40%) undergoing skull base meningioma resection. The mean age of the participants was 58 years (± 15, range 27 to 75 years), with four female patients (40%). Patients overall rated the VR-informed consent (VR-IC) positively with a mean of 4.22 (± 0.84). There was a better understanding of their pathology (mean 4.30 ± 0.92) and the planned procedure (mean 3.95 ± 1.04). Trust in the surgeon was rated with a mean of 3.47 (± 0.94). Only minimal side effects from the VR experience including dizziness or discomfort were noted (mean 4.60 ± 0.22). None of the participants dropped out of the study.

**Conclusion:**

VR-enhanced informed consent is feasible and improves patient understanding and satisfaction without significant side effects. These findings will guide the planning of a randomized controlled trial to validate the benefits of VR-IC in neurosurgery further.

**Supplementary Information:**

The online version contains supplementary material available at 10.1007/s00701-024-06355-w.

## Introduction

Informed consent (IC) is obligatory for every medical intervention and represents an important cornerstone of the patient-physician relationship and an essential procedure in current surgical practice. Especially in neurosurgery, the complexity of diagnoses and the high-risk nature of procedures make the IC of the patient crucial [7]. In addition to ethical responsibility, there are legal obligations to provide accurate and complete informed consent. Although some tasks in the clinical routine can be delegated to physician assistants or medical students, it is legally binding in Switzerland and many other countries that the patient's IC must be performed by a physician, ideally the one performing the surgery or intervention [25]. Although there are standardized written informed consent documents, the process varies considerably depending on the surgeon.

Studies have shown that many patients cannot recall most of the information provided during informed consent for surgery, especially when only verbal communication is used [7, 33]. A relevant percentage of patients are unsatisfied with the IC process in surgery, and miscommunication between surgeon and patient can lead to increased lawsuits [9, 17, 29]. In addition to potential medicolegal shortcomings, the literature indicates that a better understanding of the diagnosis and treatment process can reduce anxiety and that trust between patient and surgeon can positively influence treatment outcomes [22, 27].

Virtual reality (VR) is an emerging technology where users can move and interact within an enclosed virtual environment. Over the past few years, VR has gained relevance in various medical activities and research. The main clinical applications and research fields include surgical planning and medical education [20, 26]. So far, few publications have reported results of patient communication, such as VR-based IC. However, with an increasing demand for concepts such as shared decision making and patient-centered care, optimal IC is more relevant than ever. Recent studies have reported that patients and surgeons can benefit from using VR applied to the IC by demonstrating higher scores for objective comprehension in their VR group while having no differences in anxiety [23].

Our pilot trial focuses on the feasibility of implementing virtual reality in patient education, specifically VR-based patient-informed consent for elective neurosurgical procedures. This single-center pilot trial evaluates the feasibility and preliminary subjective perception of VR-based patient IC for elective neurosurgical procedures.

## Methods

### Patient selection

We included patients between the ages of 18 and 75 who were scheduled for elective surgical resection of skull base meningioma or clipping of intracranial aneurysm between May and December 2023. Exclusion criteria were visual or auditory impairment without adequate aid, severe cognitive impairment or psychiatric disorders, and patients who had previously received VR-based IC for surgery. All patients had received additional standard written IC before VR IC.

### Virtual reality IC

**Fig. 1:**
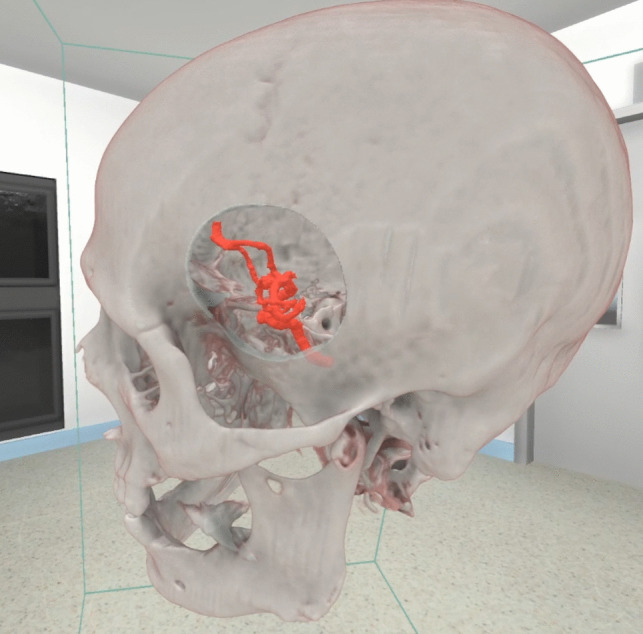
Custom virtual reality model of a patient with an intracranial aneurysm

After patient enrollment, preoperative cMRI, cCT, or digital subtraction angiography (DSA) imaging data was imported into SpectoVR (Specto Medical AG, Basel, Switzerland). Using this software, the treating surgical team created a precise virtual 3D model of the patient’s skull, including the targeted pathology, based on the 2D images (Fig. 1). VR IC was performed the day before surgery when the patient was admitted.

**Fig. 2:**
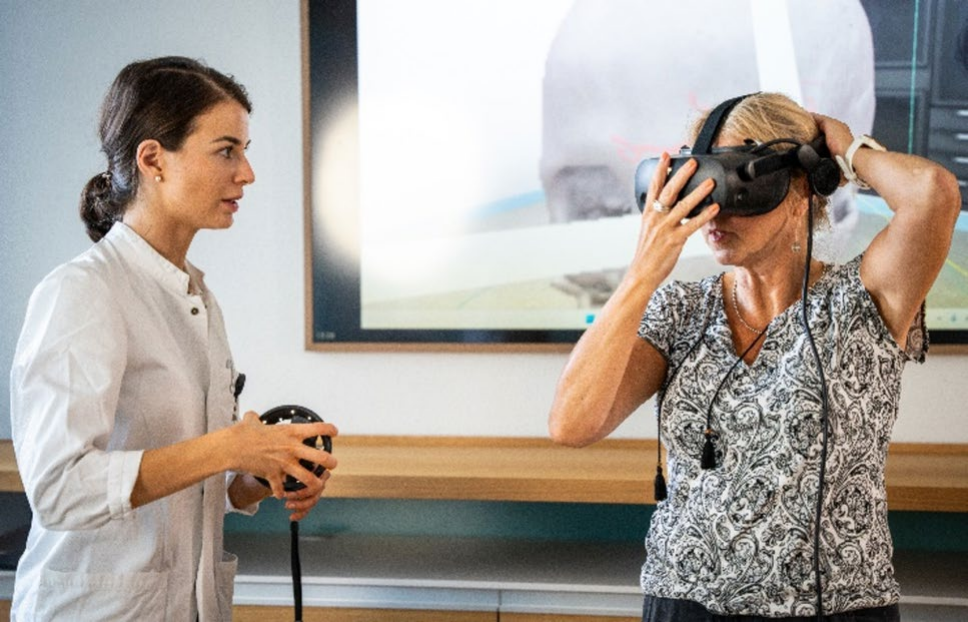
Patient-Introduction to the Virtual reality system and the 3D model

The work-flow of the VR IC was standardized, following the steps described: The surgeon first demonstrated the model and virtual reality equipment, including VR glasses and a handheld controller (HP Reverb G2 headset and HP motion controllers, HP Inc., California, United States). The user's view through the VR glasses was also mirrored onto a 2D screen. After the initial demonstration, the patient was instructed to wear glasses and move within the VR space individually under the guidance of the explaining surgeon (Fig. 2). The model itself, the anatomical position of the pathology, the virtually performed craniotomy, and the possible risks and benefits of the surgery were discussed with the patient. In aneurysm cases, different types of digitalized aneurysm clips and possible placements were demonstrated. We did not record the duration of informed consent but strictly followed the established workflow. An example of VR-based patient informed consent from our trial is shown in supplement 1.

After VR IC, patients were asked to complete a questionnaire (supplemental 1) customized for this pilot trial. The questionnaire evaluated different dimensions of patient IC and possible benefits and downsides. It included 18 questions measured on a 5-point Likert scale, one being the lowest (strongly disagree) and five the highest score (strongly agree). We categorized the questions by groups regarding trust towards surgeon and surgery (questions 1–3), understanding of the pathology (questions 4—5), understanding of the indication, risks and perioperative process (questions 6—9), the overall virtual reality experience (questions 10—13) and possible side effects of virtual reality simulation (questions 14).

### Data analysis

Descriptive statistics were presented as mean ± standard deviation and range for each individual question or grouped set of questions. They were performed using SPSS Statistics version 29, released in 2022 (IBM, New York, USA).

### Ethics

This study was conducted in accordance with the principles outlined in the Declaration of Helsinki. The research protocol was reviewed and approved by the local ethics committee (EKNZ, Project-ID: 2023–02009). All participants gave their informed consent to be included in the study.

## Results

We included a total of ten patients, of whom six (60%) underwent aneurysm clipping and four (40%) resection of a skull base meningioma. Details can be found in Table 1.

**Table 1:** Baseline characteristics

Age (Mean ± SD)	58 ± 15, Range 27 – 75
Female	*n* = 4 (40%)
Accompanied	*n* = 5 (50%)
Aneurysm Clipping	*n* = 6 (60%)
• ACOM	*n* = 3 (50%)
• MCA	*n* = 2 (33%)
• Mycotic MCA	*n* = 1 (17%)
Resection of Skull Base Meningioma	*n* = 4 (40%)
• Sinus cavernosus meningioma	*n* = 1 (25%)
• Petroclival meningioma	*n* = 1 (25%)
• Petrosal Meningioma	*n* = 1 (25%)
• Sphenoid Wing Meningioma	*n* = 1 (25%)

*SD* Standard deviation, *ACOM* Anterior communicating artery, *MCA* Middle cerebral artery

**Fig. 3:**
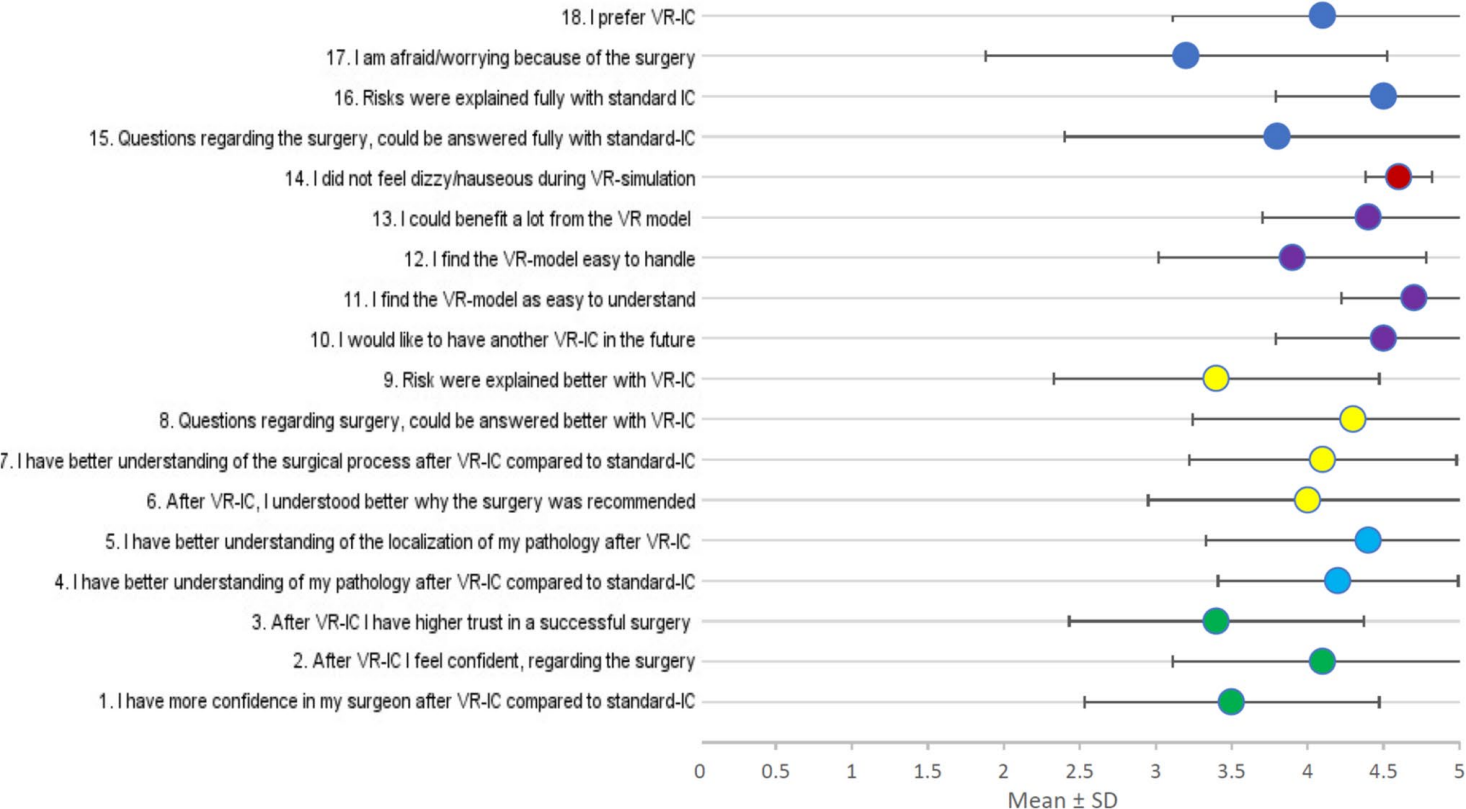
Results for each Question of the questionnaire. Color Code for Questions Groups: Green = Trust, light blue = Understanding pathology, yellow = Understanding surgery, violet = VR experience, red = Side effects, dark blue = no group. VR = virtual reality, IC = informed consent

An overview of all questions with their corresponding mean and standard deviation is shown in Fig. 3, the colors demonstrate the associated questions group. The results of each evaluated question group are shown in Table 2. We did not have dropouts in our cohort of patients.

**Table 2:** Summarized question groups

	Mean (± SD)	Range
Trust	3.47 (± 0.94)	2—5
Understanding pathology	4.30 (± 0.92)	3—5
Understanding surgery	3.95 (± 1.04)	2—5
VR experience	4.22 (± 0.84)	2—5
No side effects of VR	4.60 (± 0.22)	3—5

*VR* Virtual reality, *SD* Standard deviation

All questions related to the advantages of VR for informed consent from the patient's point of view were positively answered, with the highest mean for understanding the pathology (4.30 ± 0.92).

When analyzing the questions individually, question 15 (“I find the VR model easy to understand”) had the highest outcome, with a mean of 4.70 ± 0.48, followed by question 14 (“I would like to have VR consent again”), with a mean of 4.50 ± 0.71.

Our questionnaire also evaluated possible side effects, such as nausea or dizziness, which most patients did not experience at all (Mean 4.60, ± 0.22).

## Discussion

In this pilot trial, we evaluated the feasibility and potential benefits of the additional use of VR for IC in neurosurgery. Our study showed that using VR during IC is feasible, even in patients without prior knowledge of the technology. All patients found the VR model easy to understand, very helpful for building trust and indicated that they would prefer VR-based IC in the future. The patients only experienced negligible side effects during our trial and there were no dropouts.

Since neurosurgical procedures include complex anatomical structures that are often difficult for patients to fully understand, an optimal communication and high-quality informed consent are the basis for a solid patient-doctor relationship. Especially in the past decade, the skills of comprehensible communication and documentation have become more relevant with emerging concepts such as shared decision making and patient-centered care [1, 3, 22]. Barriers to achieving patient understanding include different levels of patient health literacy, as well as doctors' ability to communicate complex health information [15]. The sole use of written IC and verbal communication may cause comprehension gaps, and patients can often recall only small parts of the information provided throughout consent [16]. Many studies show that current verbal IC often leaves patients unsatisfied and can contribute to surgical litigations [5, 6, 9, 18].

Efforts have been made to standardize IC using uniform documents. Different methods were evaluated to improve IC by allowing patients to visualize procedures or medical conditions in a more engaging way. Hertzsprung et al. demonstrated in their RCT that patients can use stereoscopic visualization in their subjective understanding, which potentially increases patient confidence [11]. Although stereoscopic visualization can help to explain more deeply, it does not involve much more interaction for the patient. Other studies investigated the impact of customized 3D printed models in patient IC for aneurysm or skull base neurosurgery [12, 14, 19]. All studies showed that 3D-printed models were helpful and improved patient understanding. Nevertheless, the cost-value-relation for printing customized models for each IC is questionable. These attempts demonstrate the need for beneficial additions to IC that are feasible, cost-effective, and easy to implement in the clinical routine.

VR is an innovative technology that uses computer-generated environments in which users can explore and interact. It was found to be helpful for many different medical applications, such as education, surgical planning, rehabilitation, and many more [2, 26, 28, 34]. Possible benefits of VR for IC include the 3D demonstration of the patients own pathology, the ability to interact freely with the model and visualizing anatomical relations and surgical approaches. A large systematic review showed that the addition of digital technologies to informed consent has a positive effect on early comprehension without negative effects on satisfaction or anxiety [13]. Further studies showed that VR is specifically associated with better information retention compared to conventional text reading due to user involvement and reduced anxiety [21, 30, 31]. These findings are consistent with our results, which showed good subjective comprehension for VR-based PIC. Recently, Perin et al. showed that VR-based patient IC can build a better doctor-patient relationship, improve comprehension, and not increase anxiety [23]. As the first randomized controlled trial (RCT) of its kind, no standardized questionnaire was used, and comprehension was evaluated by customized individual multiple-choice questions for each patient. To truly assess the benefits of virtual reality, more RCTs are needed, with validated and standardized questionnaires.

Over the last 10 years, the number of papers addressing the benefits of VR for training medical students, residents, and even experienced neurosurgeons has increased. However, when looking closer at the current research on virtual reality in healthcare, the patient seems to be missed as a target user group. Vayssiere et al. showed in their literature research that only 5% of papers about VR in neurosurgery addressed the patient as a possible VR user [32]. Although the surgeon participates in many ways in the perioperative process, the IC is often the only access for the patient to detailed information about his surgery.

Adding new technologies to the IC could improve the relationship between surgeon and patient, patient satisfaction, and surgical outcomes [33]. The current literature can only provide preliminary evidence for proof of concept and benefits of VR-based IC [2]. Our data confirms prior findings in that the trust with the treating neurosurgeon is enhanced through the use of VR-IC.

As with any implementation of a new technology, working with VR can bear some challenges. First, the purchase of a virtual reality system adds up to additional costs, which can be a limiting factor for healthcare institutions with limited budgets. Nevertheless, in recent years, VR -systems have become increasingly more affordable [24]. With its many fields of application, investment in purchasing a virtual reality system is found to have a positive cost-value ratio [10]. Further challenges include technical limitations, such as battery life limits and image resolution [8]. However, many of these issues will be overcome with ongoing technical improvement.

Overall VR handling was subjectively perceived as easy and safe. Although VR has been reported to cause adverse effects, such as headache and nausea, which can limit its application [4], our patients reported almost no side effects. We postulate that the addition of VR to IC in neurosurgery will improve subjective patient understanding, reduce anxiety before surgery, and lead to a better patient-doctor relationship. These outcomes will be assessed in a randomized controlled trial following this pilot trial.

## Limitations

Our pilot trial included a relatively small number of only ten participants, and by design of the study, there was no control group. As only one pair of glasses and controllers was used in this study, it was not possible to interact inside the virtual environment simultaneously with the patient. Specto-VR can be used in a multi-viewer setup and this will be used in the planned RCT. We did not record the duration of informed consent but noticed that over time VR-based IC became remarkably more efficient and standardized. It must be considered that the addition of VR requires extra time for preparation, and most likely, the process of IC itself is also prolonged. The additional one-time cost of a VR system has to be considered, whereas there were no extra costs for creating the individual VR models. As there are no existing validated instruments available for this novel and innovative topic, we used a custom-developed, non-validated questionnaire. Our study provides preliminary evidence; a RCT randomizing to standard and VR-IC is planned to verify our findings.

## Conclusions

This pilot trial demonstrates that the integration of VR into the IC process for skull base meningiomas and intracranial aneurysms in neurosurgery is feasible and seems helpful in terms of patient education, trust, and satisfaction, with minimal side effects. Patients could subjectively benefit regarding understanding of their conditions and procedures and reported overall satisfaction with the VR experience. Based on the results of this pilot trial, we are currently enrolling in a subsequent randomized controlled trial.

## Supplementary Information

Below is the link to the electronic supplementary material

Supplementary file1 (PDF 224 KB)

Supplementary file2 (MP4 40837 KB)

## Data Availability

Data is provided within the manuscript or supplementary information files.
