# Supplementary material for: Virtual reality for patient informed consent in skull base tumors and intracranial vascular pathologies: A pilot study
Source: Acta Neurochir (Wien). 2024 Nov 15;166(1):455. doi: 10.1007/s00701-024-06355-w (PMC11568013; doi:10.1007/s00701-024-06355-w)
Supplement: Supplementary file 1 — Supplementary file1 (PDF 224 KB) [file 701_2024_6355_MOESM1_ESM.pdf]

	I strongly disagree	I disagree	Neutral	I agree	I strongly agree
1. I have more confidence in my surgeon after VR-IC compared to standard-IC					
2. After VR-IC I feel confident, regarding the surgery					
3. After VR-IC I have higher trust in a successful surgery					
4. I have better understanding of my pathology after VR-IC compared to standard-IC					
5. I have better understanding of the localization of my pathology after VR-IC					
6. After VR-IC, I understood better why the surgery was recommended					
7. I have better understanding of the surgical process after VR-IC compared to standard-IC					
8. Questions regarding the surgery, could be answered better with VR-IC					
9. Risks were explained better with VR-IC					
10. I would like to have another VR-IC in the future					
11. I find the VR-model easy to understand					
12. I find the VR-model easy to handle					
13. I could benefit a lot from the VR model					
14. I did not feel dizzy/nauseous during VR-simulation					
15. Questions regarding the surgery, could be answered fully with standard-IC					
16. Risks were explained fully with standard-IC					
17. I am afraid/worrying because of the surgery					
18. I prefer VR-IC					
